# 2025 List of Reviewers

**DOI:** 10.1117/1.NPh.13.1.010102

**Published:** 2026-02-15

## Abstract

Thanks to reviewers who served Neurophotonics in 2025.

*Neurophotonics* would like to sincerely thank the following individuals who served as reviewers in 2025. The success of our publication hinges on the voluntary contributions of time and energy put forth by these professionals.

Androu Abdalmalak
Nicolo Accanto
Deepshikha Acharya
Yoav Adam
Mohammed Alfadhel
Anna Letizia Allegra Mascaro
Mahnoush Amiri
Maria Arredondo
Richard Aslin
Hasan Ayaz
Wesley Baker
L. Felipe Barros
Beatrix Barth
Felix Beinlich
Addison Billing
Nadine Binder
Borja Blanco
Augusto Bonilauri
Heather Bortfeld
Marco Bove
Emily Braun
Sabrina Brigadoi
Craig Brown
Erin Buckley
Lindsay Butler
Laurianne Cabrera
Delia Cabrera DeBuc
Lin Cai
Osman Melih Can
Jiaming Cao
Stefan Carp
Shuang Chang
Jerry Chen
Shangbin Chen
Tsai-Wen Chen
Tom Cheng
Sumana Chetia
Vanessa Coelho-Santos
Davide Contini
Christian Crouzet
Carlie Cullen
Fabrice Dabertrand
Marco Dal Maschio
Ippeita Dan
Carina De Klerk
Irene de la Cruz Pavia
Ece Demir-Lira
Adam Eggebrecht
Mohamad El Amki
Morgan Fogarty
Natalie Fomin-Thunemann
Jovan Formas
Mitsuhiro (Hiro) Fukuda
Jessica Gemignani
Judit Gervain
Ariel Gilad
Louisa Gosse
Gorm Greisen
Tobias Grossman
Chenghua Gu
Eugenio Gutierrez
Daniel Hammer
Oscar Hernandez
David Highton
Fumitaka Homae
Keum-Shik Hong
Jorn Horschig
Xinlin Hou
Amy Howard
Philippe Huot
Costanza Iester
Meltem Izzetoglu
Dieter Jaeger
Sagi Jaffe-Dax
Hongbo Jia
Yali Jia
Chulmin Joo
Myeong Jin Ju
Jana Kainerstorfer
Madhuvanthi Kannan
Dimitris Kapsokalyvas
Mahesh Kate
Laura Katus
Hiroshi Kawaguchi
Karnig Kazazian
David Keays
Soheil Keshmiri
Kivilcim Kilic
Beop-Min Kim
Jeongjin Kim
Yongsoo Kim
Kassandra Kisler
Tiffany Ko
Lingjie Kong
Mariel Kozberg
Krzysztof Kucharz
Roman Kuranov
Geoff Laforge
Ana Belén Lao Rodríguez
Kijoon Lee
Talia Lerner
Frederic Lesage
Rihui Li
Chang Liu
Chao Liu
Hanli Liu
Rui Liu
Yan Liu
Shangbang Luo
Fraser MacRae
Kazuto Masamoto
Akira Masuo
Felix Matuschke
Ilaria Mazzonetto
Niall McAlinden
Rodrigo Menezes Forti
Evelyne Mercure
Daniel Milej
Bosiljka Milosavljevic
Anusha Mishra
Aseema Mohanty
Samuel Montero-Hernandez
Amreen Mughal
Timothy Murphy
Simon Musall
Laleh Najafizadeh
Kiyomitsu Niioka
Nozomi Nishimura
Ilkka Nissila
Sergio Novi Junior
Philip OHerron
Umberto Olcese
Yumie Ono
Felipe Orihuela-Espina
Boris Park
Vicente Parot
Stephen Payne
Meredith Pecukonis
Nicolas Pegard
Guy Perkins
Shaohua Pi
Paola Pinti
Laura Pirazzoli
Luca Pollonini
Robert Prevedel
Karolina Pytka
Sean Quirin
Jack Radford
Patrick Reeson
Marco Renna
Mitchell Robinson
Ravi Rungta
Saeed Samaei
Tilmann Sander-Thömmes
Hendrik Santosa
Hiroki Sato
Franca Schmid
Felix Scholkmann
Julia Scott
Ikbal Sencan-Egilmez
Li SHENG
Leena Shoemaker
Mohinish Shuckla
Juanning Si
Maheen Siddiqui
Ruth Sims
Christopher Smith
Spencer Smith
Bettina Sorger
Vivek Srinivasan
Keith St. Lawrence
Stefan Stamenkovic
Bojana Stefanovic
Aneta Stefanovska
Aleh Sudakou
Jinyan Sun
Xin Sun
Ulas Sunar
Smrithi Sunil
John Sunwoo
Margherita Tabet
Atsumichi Tachibana
Susanna Tagliabue
Sungho Tak
Toshimitsu Takahashi
Hiroyuki Takuwa
Emily Tam
Jianbo Tang
Martin Thunemann
Vladislav Y. Toronov
Alessandro Torricelli
Cam Ha Tran
Tomomi Tsunematsu
Kevin Turner
Alon Tzroya
Tara Urner
Alberto Vazquez
Vasan Venugopalan
Alexander Von Luehmann
Quan Wang
Brian White
Hannah Whitehead
Jonas Wietek
Teresa Wilcox
Philip Williams
Martin Wolf
Ursula Wolf
Kuan Cheng Wu
Melissa Wu
Dominik Wyser
Yunjia Xia
Junhua Xiao
Edward Xu
Toru Yamada
Shijie Yan
Weijian Yang
Muhammad Atif Yaqub
Mohammad Yaseen
Young-Gyu Yoon
Zhen Yuan
Mark Zarella
Qingguang Zhang
Tengyu Zhang
Xian Zhang
Yide Zhang
Hubin Zhao
Chao Zhou
Sharvari Zilpelwar
Hamoon Zohdi

